# The structural language of fungal cell wall sugars in WHO priority fungi: the determinant of pathogenicity and the gateway to new therapies

**DOI:** 10.3389/fimmu.2026.1912476

**Published:** 2026-08-10

**Authors:** Gabriel Trentin, Leonardo Martins-Santana, Carlos Pelleschi Taborda, Fausto Almeida

**Affiliations:** 1 Department of Biochemistry and Immunology, Ribeirao Preto Medical School University of Sao Paulo, Ribeirao Preto, São Paulo, Brazil; 2 Department of Biosciences and Technology, Institute of Tropical Pathology and Public Health, Federal University of Goiás, Goiânia, Brazil; 3 Laboratory of Pathogenic Dimorphic Fungi, Department of Microbiology, Institute of Biomedical Sciences, University of São Paulo, São Paulo, Brazil; 4 Centre for Medical Mycology in Latin America (CMM LATAM) Unit, São Paulo, Brazil

**Keywords:** carbohydrates, cell wall, fungal pathogens, PAMPs, targets

## Abstract

Carbohydrates, far beyond their nutritional role, are fundamental biomolecules that encode biological information and mediate critical cellular interactions. In pathogenic fungi, cell wall (CW) glycans serve as both a protective armor and a dynamic interface with the host, directly influencing virulence and immune evasion. This review discusses the intricate architecture and composition of the CW in the four WHO-critical priority fungal pathogens: *Aspergillus fumigatus*, *Candidozyma auris*, *Cryptococcus neoformans*, and *Candida albicans*. We detail how distinct carbohydrate polymers, including α- and β-glucans, and various mannans, are organized into layered structures that confer mechanical stability, modulate physicochemical properties, and display pathogen-associated molecular patterns (PAMPs). The analysis highlights species-specific adaptations: the conidial “invisibility cloak” and galactosaminogalactan layer of *A. fumigatus*; the polymorphic mannoprotein outer layer of *C. albicans*; the unique polysaccharide capsule anchored to the CW in *C. neoformans*; and the distinct mannan side chains conferring clade-specific traits in *C. auris*. Given its essentiality and absence in host cells, the fungal CW presents an ideal target for therapeutic intervention. A deep understanding of the biochemical properties and structural complexity of fungal carbohydrates provides critical insights into novel targets for therapeutic intervention, thereby contributing to the rational design of next-generation antifungals and also aiding the development of new therapies through artificial intelligence.

## Introduction

Carbohydrates are abundant organic biomolecules essential to living organisms. For many years, the biological function of carbohydrates has been neglected (1, 2), as their structure was used only to justify the stabilization and conformation of proteins and nucleic acids (3, 4). By observing cell interactions, specifically the binding between sugars and lipids/proteins on the cell membrane surface, scientists have begun to examine the role of carbohydrates in various processes (*e.g.*, cell–cell and cell–matrix interactions) and their resulting impacts (2, 3, 5–7). To evaluate the potential role of sugars, the development of elegant tools and methodologies was necessary, and the glycobiology field has arisen (2, 3). In this sense, the generic term ‘glycan’ was developed to explain the carbohydrate-based molecules, which include mono-, oligo-, and polysaccharides, and their conjugates (8, 9).

Given the considerable diversity of carbohydrates, many biological processes involve the interaction between carbohydrates and other biomolecules (10). Cell surface glycans mediate most of the host-fungal cell interaction events triggering several biological responses (6, 10–12). In this context, sugar-mediated responses are fundamental in the host-pathogen interaction as they allow the recognition and promote cell adaptation. For instance, cell-cell adhesion is crucial when leukocytes are recruited to sites of tissue inflammation, as terminal glycans mediate the rolling adhesion of leukocytes to E- and P-selectins expressed on the surface of endothelial cells (13, 14). If the carbohydrate–lectin binding process were blocked, the subsequent events of transmigration and extravasation would be interrupted, thereby preventing the inflammatory and immune responses (13, 14). The immune system recognizes sugars from pathogenic fungi via lectins expressed on the surface of macrophages and dendritic cells, thereby starting an adaptive response through intracellular signaling cascades (11, 15, 16). Cell wall (CW) provides the essential arsenal of glycans recognized by the host lectins. This structure is present as a three-dimensional armor composed of a branched network of polysaccharides, conferring fungi integrity, protection, osmotic support, and other functions (11, 15–17). Pathogenic fungi rely on the CW properties to display virulence, since this condition depends on the signaling responses stemming from the host-fungus interaction, which is mediated by the recognition of cell wall components (18). Given that, it is plausible to infer that CW recognition is intrinsically related to the development of fungal infection in hosts.

Regarding pathogenesis, the number of fungal infections has continually increased, with a significant impact on human health (19). Recently, the World Health Organization (WHO) published a priority list of pathogenic fungi that affect human health. Four species are highlighted as being critical priority: *Aspergillus fumigatus*, *Candidozyma auris*, *Cryptococcus neoformans*, and *Candida albicans* (20). The causes of this increase are multifactorial, encompassing climatic, socioeconomic, genetic, molecular, antifungal resistance factors, and others (19, 21, 22). Molecular modifications in protein, CW structure, and biofilm composition increase resistance to available antifungals and allow pathogenic fungi to evade the immune system, thereby increasing the difficulty of treatment in such inflammatory scenarios (23–28).

In general, the fungal CW is composed of two distinct layers, spatially arranged to display different functions. The inner layer, mostly composed of intermolecularly interacting chitin and glucans, functions as a less diverse backbone core in which other proteins and biomolecules might anchor. These features enable the inner layer to display functions related to shape and strength (29, 30). In turn, the outer layer is the most chemically diverse among fungal species. Mannosylated biomolecules are prevalent in this layer, which confers functions related to hydrophobicity, adhesion processes, and, finally, distinct immunological properties (30). Given the complex organization of the outer layer, the compositional structure of this wall may vary significantly in fungal species, which can modulate inflammatory processes in the host-fungi interaction.

In this review, we address the structures and features of cell wall carbohydrates of the WHO priority fungal pathogens (*A. fumigatus*, *C. auris*, *C. neoformans*, and *C. albicans*). We aim to summarize key observations from years of research, highlighting the structure and architecture of the cell wall as potential targets for new antifungal therapies, as well as how new in-silico-based approaches could contribute to the search for new fungal target candidates for drug development or diagnostic methods.

## The fungal cell wall is a source of pathogen-associated molecular patterns

A medieval warrior’s capacity to endure the rigors of military combat was dependent upon his suit of heavy, resistant metal armor (31). Unlike the armor of medieval soldiers, the fungal CW is a dynamic natural armor composed of various sugars, with its composition varying across species (17, 30). The fungal CW is a three-dimensional structure that influences the fungal life cycle (17, 29, 30). In pathogenic fungi, CW plays a dual role as the shield and the sword, interacting with the host environment not only to protect fungal cells, but also aiming to establish viable conditions for these cells to survive (17, 29).

The fungal CW is composed of over 90% polysaccharides, and across different pathogenic species, a variety of branched carbohydrates allow for the formation of a sugar network (36). The architecture of the CW consists of two layers presenting distinct compositions. The unexposed layer, which consists of a network of glucan polymers and N-acetylglucosamine derivatives (*e.g.*, chitin, chitosan), is primarily structural, providing cellular integrity, shape, and elasticity (29). The exposed layer, in contrast, is composed of diverse polysaccharides and glycosylated proteins that govern the fungus’s relationship with the extracellular environment (29, 32, 33). Every aspect of this three-dimensional scaffold plays an important role in stabilizing the whole structure, impacting its physicochemical properties (*e.g.*, malleability, elasticity, adhesion, charge, hydrophobicity, porosity, permeability) (29). Several polysaccharides of the fungal CW are pathogen-associated molecular patterns (PAMPs) that are recognized by the immune system cells (**Table 1**) (29, 33, 34). Immune cells identify fungal pathogens via interaction with the Pattern Recognition Receptors (PRRs), such as toll-like receptors (TLRs), Nucleotide-binding and Oligomerization Domain-like receptors (NODs), mannose receptors (MRs), Dendritic Cell-Specific Intercellular adhesion molecule-3-Grabbing Non-integrin (DC-SIGN), and others (29, 33, 34). Notably, despite the complex composition of the CW, it is not static; stress from the host milieu triggers CW remodeling, resulting in the shaving or masking of some PAMPs. These alterations may allow the fungus to survive and evade the immune response (35).

**Table 1:** Innate immune receptors and fungal carbohydrate ligands.

Immune cells PRRs	Localization	Fungal PAMPs	References
Dectin-1	Cell surface	β-glucans	(161)
Dectin-2	Cell surface	Mannose	(162)
DC-SIGN	Cell surface	Mannose and mannosylated proteins	(163)
MR	Cell surface	N-linked mannans and chitin	(164)
TLR2/TLR4 TLR2/TLR1 TRL2/TLR6	Cell surface	GXM	(165, 166)
TLR 2	Cell surface	α/β-glucans, mannosylated components, chitin, GXM and phospholipomannans	(167–169)
TLR4	Cell surface	O-linked mannans	(85)
TLR9	Endosome	Chitin	(170)

## From recognition to response: downstream signaling, host-cell diversity, and immune evasion at the molecular level

PRR engagement by CW glycans is only the first step of a signaling cascade that determines the quality, magnitude, and polarization of the antifungal immune response. Dectin-1, upon binding exposed β-1,3-glucan, phosphorylates its cytoplasmic hemi-immunoreceptor tyrosine-based activation motif (hemITAM), recruiting and activating spleen tyrosine kinase (Syk); Dectin-2 and Mincle, which lack signaling motifs of their own, instead associate with the Fc receptor γ-chain to trigger the same Syk-dependent cascade upon mannan recognition (36–38). Syk activation converges on the CARD9–Bcl10–MALT1 (CBM) complex, which drives NF-κB and AP-1 nuclear translocation and the transcription of pro-inflammatory cytokines (TNF-α, IL-6, IL-23) and chemokines, ultimately licensing Th17 differentiation, a T-cell program central to mucosal and cutaneous antifungal defense (36, 37, 39).

In parallel, Dectin-1/Syk signaling primes and activates the NLRP3 inflammasome, in some contexts through a non-canonical caspase-8-dependent route, resulting in caspase-1 activation and maturation of IL-1β and IL-18, cytokines that amplify neutrophil recruitment and Th17 responses (38). TLR2 and TLR4, engaged by phospholipomannans, O-linked mannans, and glucuronoxylomannan (GXM), signal mainly through MyD88 to activate NF-κB and IRF-family transcription factors, while endosomal TLR9 senses fungal chitin fragments and DNA motifs (15, 29, 33). These pathways are not redundant but cooperative: co-engagement of Dectin-1 and TLR2, for instance, synergistically amplifies cytokine output compared with either receptor alone, illustrating how the layered CW architecture is decoded as a combinatorial signal rather than a single input (11, 15).

The relative contribution of each PRR–pathway pair, however, differs markedly among the four WHO-priority pathogens, reflecting their distinct CW architecture (**Table 2**). In *A. fumigatus*, resting conidia are largely silent to Dectin-1 because the rodlet/melanin coat masks β-glucan; germination exposes β-1,3-glucan and α-1,3-glucan on swollen conidia and hyphae, triggering Dectin-1/Syk/CARD9 signaling and robust neutrophil-dependent killing, while galactomannan and galactosaminogalactan (GAG) additionally engage TLR4 and modulate the response (29, 32, 40). In *C. albicans*, the yeast-to-hyphae transition switches the dominant PRR usage from Dectin-2/Dectin-3 (recognizing α-mannan on yeast) toward Dectin-1 and TLR2 (as β-glucan becomes exposed on hyphal septa and scars), a shift that shapes whether the response favors containment or a pro-inflammatory, tissue-damaging phenotype (39, 41). In *C. neoformans*, the GXM/GXMGal-rich capsule largely shields β-glucan and chitin from Dectin-1 and TLR9, so recognition depends disproportionately on TLR2/TLR4 and mannose receptor engagement by capsular polysaccharide, a pathway that is comparatively weak at driving protective Th1/Th17 responses and can instead favor a tolerogenic, IL-10-biased outcome (15, 29, 42). In *C. auris*, the α-1,2-mannose-phosphate side chains that distinguish its clades differentially engage Dectin-2 and MR, and cell-wall mannosylation impairs neutrophil-mediated killing through pathways mechanistically distinct from those used by *C. albicans*, underscoring that even structurally similar mannans can be decoded differently depending on their side-chain chemistry and clade-specific presentation (43–45).

**Table 2:** Downstream signaling pathways and functional outcomes of major PRRs engaged by fungal CW glycans.

PRR	Adaptor/kinase	Downstream pathway	Main functional outcome	References
Dectin-1	Syk → CARD9-Bcl10-MALT1	NF-κB/AP-1; non-canonical NLRP3/caspase-8 inflammasome	TNF-α, IL-6, IL-23, IL-1β; Th17 priming; phagocytosis	(11, 15, 36, 38)
Dectin-2/Dectin-3	FcRγ-Syk → CARD9	NF-κB; NFAT	Th17 priming; synergizes with TLR signaling	(37, 39, 162)
Mincle	FcRγ-Syk → CARD9	NF-κB	Pro-inflammatory cytokine production	(36, 37)
MR/DC-SIGN	Raf-1-dependent (non-canonical)	Modulation of NF-κB acetylation	Fine-tunes cytokine output; antigen uptake	(163, 164)
TLR2/TLR4	MyD88-TIRAP	NF-κB, IRF	Pro-inflammatory cytokines; cooperates with Dectin-1	(15, 29, 85, 165–169)
TLR9	MyD88 (endosomal)	NF-κB, IRF7	Recognition of chitin/fungal nucleic acid motifs	(170)

These recognition events are also profoundly shaped by the responding cell type. Macrophages rely heavily on Dectin-1/CARD9-driven phagocytosis and cytokine production, and their polarization state (M1 versus M2-like) determines whether engulfed conidia or yeasts are efficiently killed or instead persist intracellularly, as documented for *A. fumigatus* conidia and *C. neoformans* yeasts within macrophage phagosomes (11, 40). Neutrophils, in contrast, combine Dectin-1/CR3-mediated phagocytosis with oxidative burst and the release of neutrophil extracellular traps (NETs) that entrap both yeast and hyphal morphologies; *A. fumigatus* GAG subverts this response by inducing neutrophil apoptosis and dampening NET formation, while *C. auris* mannosylation impairs neutrophil killing through a route distinct from *C. albicans* (39, 45, 46). Epithelial cells, which form the first physical barrier at mucosal surfaces, express Dectin-1, TLR2, TLR4, and Epidermal Growth Factor Receptor (EGFR), and mount a biphasic MAPK response that discriminates yeast from hyphal morphology, favoring chemokine secretion (*e.g.*, CXCL8/IL-8) and antimicrobial peptide release over direct microbicidal killing (47–49). This cell-type compartmentalization means the same glycan can generate distinct, sometimes opposing, functional outcomes depending on whether it is sensed by a phagocyte poised for killing or by a barrier cell oriented toward alarm signaling.

Collectively, these observations show that immune evasion by WHO-priority fungi operates mechanistically at the level of signal input rather than simply “hiding” from the immune system: rodlet/melanin and capsule layers physically occlude high-affinity PAMPs from Syk-coupled receptors, GAG actively subverts neutrophil effector function, and clade- or morphotype-specific remodeling of mannan side chains redirects recognition toward PRR–pathway combinations that favor fungal persistence over pathogen clearance. Understanding these mechanisms in a species- and cell-type-resolved manner is therefore not only of fundamental immunological interest but also informs which PRR–pathway axis might be therapeutically exploited or restored (*e.g.*, via glucan unmasking or CARD9 pathway agonism) as an adjunct to conventional antifungal therapy.

## Aspergillus fumigatus

**Figure 1:**
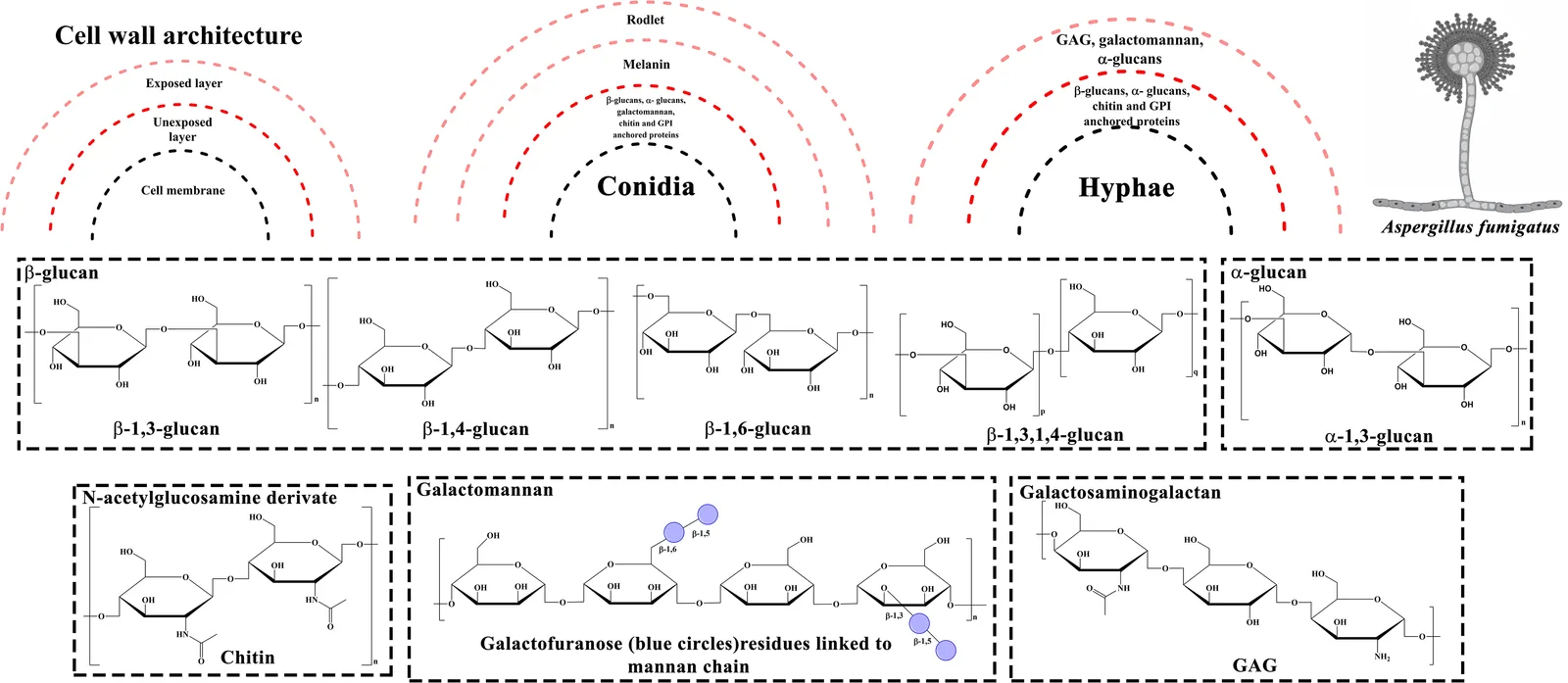
*Aspergillus fumigatus* cell wall architecture. The diagram highlights the carbohydrate structure in two morphotypes: conidia and hyphae. The exposed layer is represented by light red dashed lines, while the unexposed layer is shown in dark red. Note the presence of GAG, galactomannan, and α-glucan in the hyphae.

To survive in a host environment or reproduce, *Aspergillus fumigatus* produces conidia, which then germinate into hyphae, triggering invasive aspergillosis in immunocompromised patients (50). Both conidia and hyphae have distinct CW composition and architecture (**Figure 1**) (18). The dormant conidial CW is organized into two distinct layers: an inner polysaccharide skeleton of α-/β-glucans and chitin, and an outer layer containing rodlet proteins and melanin (51, 52). In this review, we will focus on only two morphotypes: resting conidia and hyphae.

The dormant conidial outer layer provides an invisibility cloak that conceals the conidia from immune system cells (51, 52). Combined, the rodlet layer and melanin are responsible for the hydrophobic properties of conidia and their buoyancy in the air (52). The deletion of the gene that encodes the RodA protein, which resulted in the removal of the rodlet layer and an increase in hydrophilic behavior, changed the morphology and induced a host immune response against *A*. *fumigatus* (53). Also, knockout strains for the genes related to melanin biosynthesis (*pksP*, *ayg1*, and *arp2*) altered the composition of the CW and conidial rigidity, as well as induced the maturation of human dendritic cells (54). Rodlet- and melanin-deficient conidia triggered a pro-inflammatory response in human monocytes, suggesting the immune role of the exposed layer in covering the conidia from the immune system (53). The removal of components of the outer layer impacted the physicochemical properties of the conidia surface and altered the CW architecture, exposing the inner layer (54).

The resting conidial inner layer is composed of a branched network of α- and β-glucans, mannan, and chitin (33, 52, 53). Due to high amounts of pigments and/or proteins, the investigation of conidial CW structure is a challenge (55). The composition of the unexposed layer is different from the hyphae (55). The main component of the conidial CW is β-glucan, accounting for nearly 50% of the total sugar quantification, while mannan, chitin, and α-glucans were also identified (55). The architecture of the inner layer is structured by a main amorphous scaffold of β-1,3-glucan cross-linked with α-1,3-glucans and chitin. A layer of chitin covers the plasma membrane (56, 57). Once the inner layer is exposed, β-glucans are recognized by immune system cells, triggering an immune response (58).

The conidial germination is triggered by external signals, leading to several morphological alterations (59). The CW architecture is remodeled during the progression of germination, a process in which the rodlet layer is lost and hydrophobicity decreases, thereby allowing for the emergence of α-1,3-glucan and adhesive properties (52). The hyphal CW core is a cross-linked network composed of a main branched β-1,3/1,6-glucan scaffold with a high number of nonreducing ends covalently attached to chitin, galactomannan, and β-1,3/1,4-glucan (60–62). While the composition of the unexposed layer is similar in conidia and hyphae, the outer layer of hyphae is composed of α-glucans, galactomannan, and galactosaminogalactan (GAG) (58, 60, 63). However, due to the complexity of the covalent linkages and the spatial arrangement of molecules within the CW, the organization of this layer remains unclear (64). It is important to highlight the presence and role of GAG in the CW. GAG, a heteropolymer composed of galactopyranose, galactosamine, and N-acetylgalactosamine linked by α-(1,4) linkages, surrounds the other CW layers and plays an important role during the progression of *A. fumigatus* infection (58, 63, 65).

The biological properties of GAG depend on more than its monosaccharide composition. Initial biochemical analyses of GAG produced under defined culture conditions indicated that the galactosamine residues in the material analyzed were N-acetylated, with no evidence of deacetylated galactosamine (66). Later studies showed that GAG undergoes an additional maturation step mediated by Agd3, an enzyme that partially removes acetyl groups from the polymer (67, 68). Agd3-dependent modification generates positively charged galactosamine-rich regions, favoring GAG retention at the *A*. *fumigatus* hyphal surface and contributing to biofilm formation and virulence (67, 68).

The distinct immune effects reported for GAG may therefore reflect differences in its maturation state, structural properties, and biological context. GAG has been linked to reduced inflammatory activation through mechanisms involving IL-1 receptor antagonist and decreased Th1- and Th17-associated cytokine responses (69). However, deacetylated GAG can also be recognized through host pathways involving the NLRP3 inflammasome, contributing to antifungal responses against *A. fumigatus* (70). Moreover, the anti-inflammatory activity of GAG-derived oligosaccharides is influenced by de-N-acetylation and oligomer size (71). Therefore, how GAG maturation, structural heterogeneity, and biological context shape host immune responses remains not fully understood.

## Candida albicans

**Figure 2:**
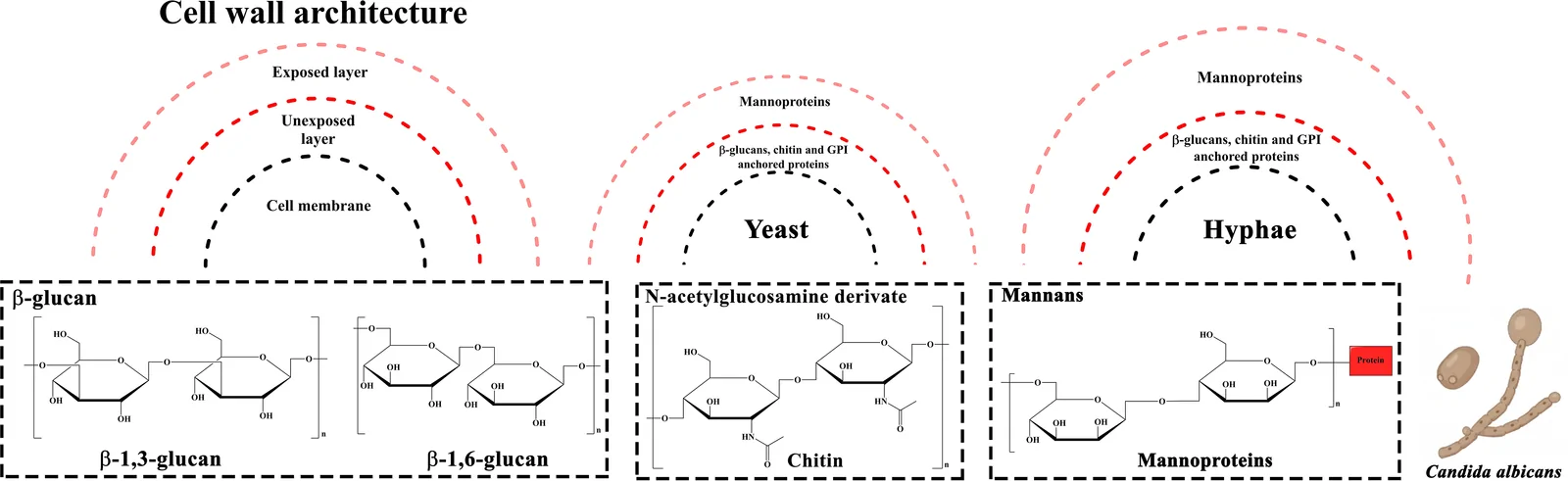
*Candida albicans* cell wall architecture. The diagram illustrates the carbohydrate structure in yeast and hyphae. The exposed layer is represented by light red dashed lines, while the unexposed layer is shown in dark red. Note the expanded exposed layer in the hyphal form.

*Candida albicans* is a polymorphic fungus that exists in distinct morphologies: yeast, pseudohyphae, and hyphal form (72). The pathogenesis in this species depends on environmental changes, thereby initiating candidiasis particularly in immunocompromised individuals (19, 34, 73). *C. albicans* is divided into two main serotypes, A and B, according to differences in cell-surface mannan epitopes. The serotype A mannan contains additional β-1,2-linked mannose residues in the acid-stable region, while serotype B lacks or contains lower levels of these antigenic structures (74–76). The CW of *C*. *albicans* is composed of approximately 90% carbohydrates and 10% protein, being divided into two layers (**Figure 2**) (34, 77). The hyphae’s outer layer is mainly composed of O- and N-mannans linked to CW proteins, playing an important virulence role in the fungal-host interaction (75, 77). Specifically, N-glycans have a main α-1,6-mannan backbone with side chains containing α-1,2-, α-1,3-, and β-1,2-mannose (75). There are three types of β-1,2-mannose linkages: (i) a sugar-phosphate bond via a phosphate group, common in serotypes A and B; (ii) and (iii) sugar–sugar bonds to α-1,2- or α-1,3-mannose residues on the side chain, respectively, both of which are common in serotype A (75). The exposed layer plays an important role during candidiasis infection; its hydrophobic properties, which are associated with the carbohydrate-protein architecture, allow for adhesion to the epithelium and can modulate the immune response (76, 77).

The inner layer is primarily composed of chitin, and β-glucans, arranged in a network structure (78). Microfibrillar chitin is formed by anti-parallel chitin homopolymers linked by hydrogen bonds; this structure is, in turn, linked to β-1,3/1,6-glucans and glycosylphosphatidylinositol (GPI) anchors (78, 79). The malleability of the *C. albicans* CW is attributed to the helical conformation (triple and single helices) of β-1,3-glucan, which extends across the wall architecture to the outer mannan layer (78). Although the mannan layer is one of the main components of the exposed layer, mannoproteins might be anchored to the inner layer. O-mannan conformation acts as a stabilizer for the exposed layer; it is anchored to GPI proteins by the carboxy-terminal end, while the N-mannan binds to the amino-terminal end (29, 78). As the carbohydrate and protein composition of the yeast and hyphal forms of *C. albicans* differs substantially, differences in the exposure of these molecules to the extracellular environment and their interactions with host immune cells are highly relevant to fungal development and host–fungus interactions (77). β-glucan visibility at the *C. albicans* surface is not fixed, but varies according to infection stage, morphotype, stage of hyphal development, host-associated cues, and antifungal treatment (80–83). The regulatory mechanisms that determine when β-glucan becomes accessible at the cell surface remain only partly resolved (82), indicating that β-glucan should not be viewed simply as an inner-wall component that is always hidden from the host (80, 81). Morphogenesis also alters the repertoire of surface proteins, including the hypha-associated adhesin Als3, which contributes to adhesion and host cell interactions (84). Alterations in CW ligand exposure can influence immune recognition, as host sensing of *C. albicans* involves receptors that detect both mannan- and β-glucan-containing structures, including lectin receptors and Toll-like receptors (85). Together, morphotype-dependent changes in polysaccharide exposure and surface protein expression reshape the host-facing fungal surface and contribute to immune modulation.

## Cryptococcus neoformans

*Cryptococcus neoformans* is a pathogenic fungus that infects immunocompromised patients (*e.g.*, HIV infected individuals) by inhalation of yeast, which can differentiate into cells with different morphotypes (86, 87). During infection, a heterogeneous population of *C. neoformans* cells, ranging in size from less than 10 μm to 100 μm, can be found in infected tissues (88). Titan cell formation is influenced by host-associated conditions but depends on fungal signaling pathways for its execution. Host factors, including immune context and environmental cues, can promote titanization, whereas fungal regulators such as the Gpr/cAMP/PKA/Rim101 pathway control the ability of *C*. *neoformans* to undergo this morphological transition (89–91). The interplay between host cues and fungal regulation may help explain why titan cell formation varies among clinical isolates.

**Figure 3:**
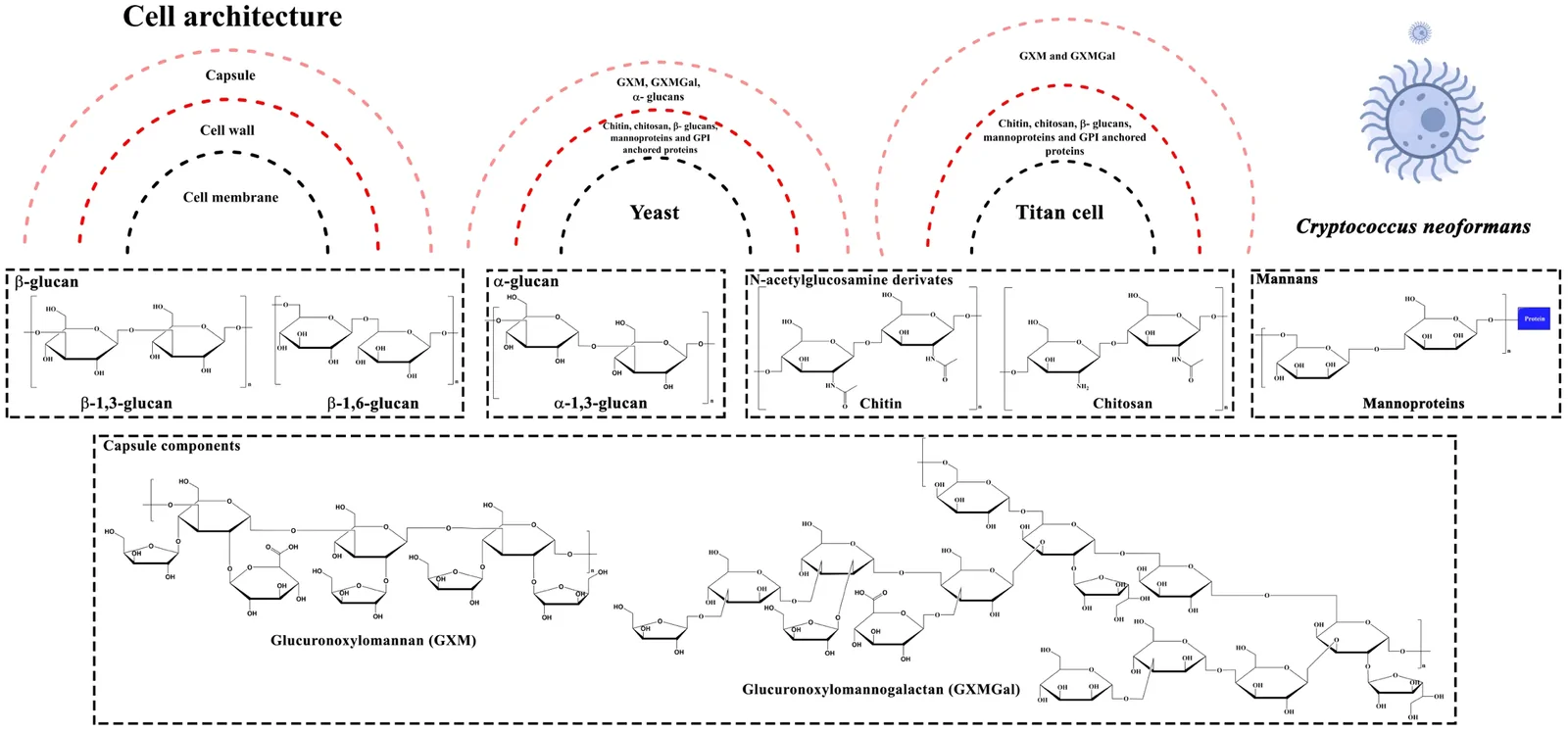
*Cryptococcus neoformans* cell wall architecture. The diagram illustrates the carbohydrate structure in yeast and Titan cells. The exposed layer is represented by light red dashed lines, while the unexposed layer is shown in dark red. Note the enlarged layers and the exclusive presence of GXM and GXMGal in the Titan cells.

*C*. *neoformans* isolates are classified into serotypes based on biochemical differences of their capsule polysaccharides (92). Serotypes A and D correspond to *C. neoformans* var. *grubii* and *C. neoformans* var. *neoformans*, respectively, and hybrid AD strains have also been reported. These serotypes are mainly distinguished by antigenic and structural differences in the capsular polysaccharide GXM, including variations in xylose substitution, O-acetylation and GXM motif distribution (93–96). To simplify, here, we will use *C. neoformans* to describe the common features of CW and capsule in yeast (also known as typical cells) and titan cells, giant cells with a robust CW disposition that confer resistance to immune defenses, increasing the virulence potential of this species. The CW architecture is a complex network composed of β-1,3/1,6-glucans, α-1,3-glucan, chitin, chitosan (the deacetylated form of chitin), mannoproteins, and GPI-anchored proteins (88, 97, 98). In general, the *C. neoformans* CW is also divided into two layers; the inner layer is arranged parallel to the cell membrane and is composed of β-1,3/1,6-glucans crosslinked to chitin and its deacetylated form, chitosan, which stabilizes the cell wall via hydrogen bonding (**Figure 3**) (88, 97–99). The outer layer is mainly composed of α-1,3-glucan and β-glucan. Of these, α-1,3-glucan plays an important role at the outer layer’s surface, being responsible for anchoring the entire capsule structure (18, 88, 97, 98).

A particular feature of this species is the occurrence of the capsule, a three dimensional structure adjacent to the outer layer of the CW. The *C. neoformans* capsule is a unique, complex structure and can be present in culture supernatant in exopolysaccharide form or attached to the cell (98, 100). It is the primary virulence factor associated with cryptococcal infection, and its antigens are used for diagnosis (98, 101). The capsule is composed of three constituents in different proportions (98). The most abundant is glucuronoxylomnan (GXM), which comprises approximately 90% of the capsule’s total composition. It is formed by a trisaccharide α-1,3-mannose backbone and three different sugar side-chains, such as β-1,2/1,4-xylopyranosyl and β-1,2-glucopyranosyluronic acid (102). GXM is a heteropolymer, and the variable arrangement of xylose and glucuronic acids on its branches allows for different crosslink motif combinations (101). The other constituents are glucuronoxylomannogalactan (GXMGal, also known as GalXM) and mannoproteins (103, 104). GXMGal is also a heteropolymer structure formed by α-1,3/1,4-mannose, β-1,2/1,3-xylopyranosyl, and β-glucopyranuronosyl acid linked to an α-1-6-galactopyranose (and β-galactofuranose, in small amount) backbone (105). Nevertheless, there are two main types of variation in the GXMGal structure: first, the presence of variable groups on the side chains (including β-xylopyranosyl, β-glucopyranuronosyl acid, and O-acetyl groups on β-galactopyranosyl), and second, variation in the galactose backbone (101, 105). Both GXM and GXMGal, which has a highly diverse structure, present immunological properties (101, 106).

To understand its multifaceted role, the CW and capsule components of *C. neoformans* have been investigated. α-1-3-glucans, for instance, are essential for maintaining capsule attachment to the CW (107). Disruption of the α-glucan synthase gene (*ags1*) resulted in CW malformation, which was accompanied by a compensatory mechanism involving morphological alterations, increased chitin/chitosan levels, and β-glucan redistribution (98, 107). Moreover, β-glucans are important elements in the maintenance of the CW architecture (18). Disruption of genes related to β-glucans (*kre5*, *kre6*, and *skn1*) induced alterations in CW architecture and chitin accumulation, leading to reduced virulence in those strains (98, 108). The flexible polymer chitosan may play an important role in both cell wall plasticity and the dissociation of budding cells from mother cells (109). Furthermore, during vegetative growth, the deletion of genes encoding chitin/polysaccharide deacetylases resulted in a decrease in CW chitosan levels and the exposure of melanin to the extracellular milieu (109). The capsule components are specific to *Cryptococcus* species associated with virulence (98, 101). The *Cap* gene family encodes proteins involved in capsule biosynthesis. Consequently, the deletion of these genes results in an acapsular phenotype (110, 111). Furthermore, signaling pathways, including the high osmolarity and cAMP/protein kinase A pathways, play a role in modulating capsule biosynthesis allowing *C. neoformans* to resist host defences (110).

As aforementioned, there are a variety of morphotypes during cryptococcal infection (86). The titanization process allows *C*. *neoformans* to adapt and survive by increasing the cell size and the thickness of the CW (86, 112). Titan cells prevent phagocytosis by host cells due to their increased size (100, 112). The composition of the CW and capsule in titan cells is different from that of yeast cells (100). Monosaccharide quantification revealed differences in the composition of the CW and capsule components between titan cells and yeast cells, with xylose, glucosamine, and mannose being more abundant in the latter (100). The thickening of the capsule and the cell wall in titan cells increases the presence of capsule components, mannan fibrils, and glucosamine derivatives, which could be related to immune evasion and disease persistence (86, 100).

## Candidozyma auris

*Candidozyma auris* (formerly known as *Candida auris*) is an emerging antifungal-resistant pathogenic yeast reported across five continents (113–115). A recent phylogenomic reclassification of the *Candida auris–Candida haemuli* clade proposed the transfer of *Candida auris* to the genus *Candidozyma* (114). Therefore, throughout this review, we use the nomenclature *Candidozyma auris* in accordance with the proposed taxonomic reclassification.

Initially, reports on *C. auris* described it exclusively as a yeast, while also highlighting its virulence (116). However, filamentation has been observed in clinical isolates under stress or inducing conditions (115, 117, 118). In *C. auris*, alternative growth forms have been reported under specific conditions, including a persistent filamentous phenotype after mammalian passage and pseudohyphal development in response to genotoxic stress (115, 117). Filamentous forms have also been observed in clinical isolates from different *C. auris* clades (118), although their contribution to human infection remains unclear. Beyond its morphological plasticity, *C*. *auris* has demonstrated remarkable environmental adaptation, displaying unique features that differ from those of other *Candida* species, including thermotolerance and osmotolerance (119). The high thermotolerance of *C*. *auris*, together with evidence of its occurrence in natural environmental niches, has led to the hypothesis that adaptation to increasing environmental temperatures may have contributed to its emergence as a human pathogen (119–122). Environmental adaptability may also be reflected in CW plasticity, as environmental stressors can modulate CW composition and thereby influence immune evasion (43).

**Figure 4:**
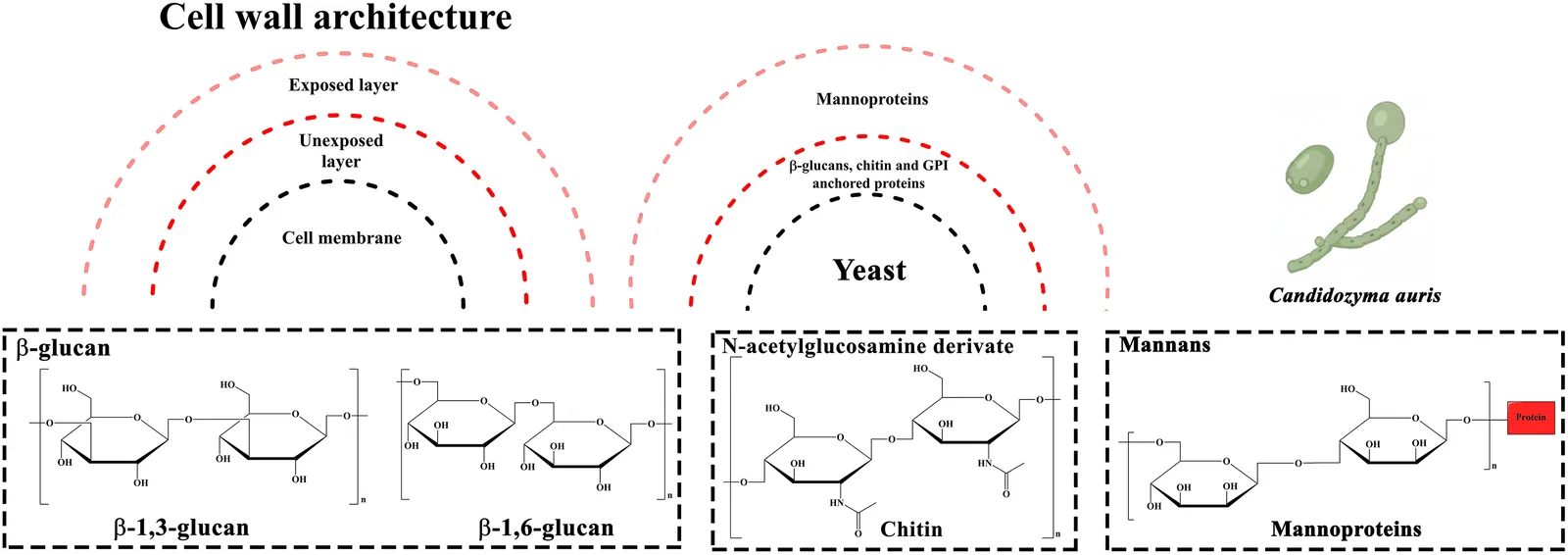
*Candida auris* cell wall architecture. The diagram illustrates the carbohydrate structure. The exposed layer is represented by light red dashed lines, while the unexposed layer is shown in dark red. Note that the cell wall composition is described only for the yeast form.

The *C. auris* CW is divided into the mannosylated proteins outer layer and to the α-1,3/1,6-glucans, β-1,3/1,6-glucans and chitin inner layer (**Figure 4**) (44). Mostly, the *Candida* spp. yeasts display glycosylphosphatidylinositol (GPI) anchored to mannoproteins as a common feature for post-translational modification. In the fibrillar outer layer, mannoproteins contain O-linked mannan (O-mannan and O-1,2-phospholipomannan) as well as side-chain N-linked mannans (comprising α-1,2/1,3/1,6- and β-1,2-mannopyranoside residues) (43). However, *C. auris* possesses an α-1,2-mannose-phosphate side chain attached to an α-1,6-N-mannan backbone, a specific feature of its cell wall that is responsible for the differences between clades (43, 123). The side chains maintain the attachment of the outer layer to the inner layer (124). The *C. auris* inner layer is similar to that of *C. albicans*, featuring a conserved architecture formed by the (physical or chemical) interaction of chitin and α-1,3/β-1,3-glucans (43, 124). On the other hand, the β-1,6-glucans confer mobility and flexibility to the inner layer (124).

There are strain-specific molecular and genetic features associated with *C. auris* isolates (124, 125). Variations in the main CW components, such as mannan, chitin and glucan, have been observed among different clades (43). These variations may contribute to adaptation within host niches and influence antifungal responses (43). Antifungal resistance in *C. auris* involves distinct mechanisms across drug classes. Fluconazole resistance has been directly associated with mutations in *ERG11*, while alterations in *TAC1B* can elevate *CDR1* expression and contribute to reduced fluconazole susceptibility in clinical isolates (126, 127). Echinocandin resistance is strongly associated with mutations in *FKS1*, which encodes the catalytic subunit of β-1,3-glucan synthase (128). Beyond these genetically defined resistance mechanisms, echinocandin exposure can induce adaptive CW changes in *C. auris*, with increased β-1,6-glucan forming part of a response that helps preserve wall stability under drug stress (124).

Antifungal-induced CW remodeling may extend beyond the maintenance of structural integrity by altering polysaccharide accessibility at the cell surface. The extent of β-glucan exposure in *C. auris* therefore appears to be highly context-dependent. Host-relevant environmental cues and antifungal treatment can modify the accessibility of β-glucan at the *C. auris* surface, affecting how fungal cells interact with macrophages and neutrophils (129, 130). Mannan-dependent masking appears to support immune evasion in *C. auris*, as disruption of the outer mannan-rich layer increases exposure of immune-stimulatory wall components and promotes stronger neutrophil recognition, uptake, and antifungal activity (45). Nevertheless, the mechanisms regulating β-glucan exposure across different genetic backgrounds and host-associated conditions remain incompletely understood.

## The fungal cell wall as a source of vaccine candidates

Given the diversity of chemical components of the fungal CW, especially those associated with the outer layers, this structure represents a rich source of biomolecules that can be used as potential antigens in the development of vaccines against fungal infections.

Literature provides some notable approaches that can be discussed regarding their relevance and promising character. For instance, Singh et al. (2022) demonstrated that the immunization with antibodies targeting the cell wall proteins Als3 and Hyr1 protected mice from neonatal candidiasis (131). Moreover, Hester et al. (2022) showed that in a model of mice cryptococcosis, survival rates were improved by cross-reactivity between antigens from the chitin deacetylase family (132). These enzymes are particularly important because they recognize GlcNAc patterns in chitin and metabolize it into chitosan through deacetylation, thereby contributing to the dynamism of the fungal cell wall (133). Notably, chitin deacetylases do not share significative homology to human proteins, enabling them to be used as antigens in vaccine development (134).

Moreover, Crawford et al. (2024) demonstrated that targeting the GXM molecules of the capsule of *C. neoformans* through semisynthetic glycoconjugate vaccine candidates could induce antibody production in mice, highlighting the potential of *C. neoformans* capsule in harboring target molecules capable of provoke immune response (135).

Notwithstanding, glucan particle-delivered vaccines represent an elegant way to induce immunogenicity against fungal components. Specht et al. (2017) and Hester et al. (2020) showed that recombinant *Cryptococcus* antigens, such as chitin deacetylases, delivered by glucan particles, induced protection and prolonged survival in mice (136, 137).

More recently, Specht et al. (2022) have addressed the role of chitin deacetylase antigen character and the ability of the host to recognize it through prediction binding to the major histocompatibility complex class II (MHC II) in mice (138). The authors showed that a glucan particle-delivered peptide sequence predicted to interact efficiently with MHC H2-IAd allele complex found in BALB/c mice could induce protection against cryptococcosis. Notably, mutations in the peptide predicted to attenuate MHC II interaction impaired the protection after vaccination. This suggests that not only are glucan particles efficient vehicles to deliver vaccines, but also cell wall-remodeling enzymes are an elegant strategy to induce immunogenicity against fungal infections (138).

In this sense, it seems plausible to mention the critical role of the adaptive immunity in conferring protection after immunization. Wang et al. (2023) showed that CD4^+^ T lymphocytes are crucial for eliciting protection induced by vaccines composed of glucan particles associated with chitin deacetylase proteins in mice, reinforcing the significant role of the cell wall components in aiding to modulate immunogenicity by recruiting the adaptive immunity in cryptococcosis (134).

## Same bricks, distinct walls: how do WHO priority fungi orchestrate their army?

The fungal CW is frequently described using a simplified layered model based on the relative exposure of polysaccharides and other CW-associated molecules to the external environment (100, 124, 139). The outer layer is generally associated with host interactions and immune evasion, whereas the inner layer is primarily involved in protection and structural integrity and is composed of an extensive network of β-glucans and chitin (18). Although these carbohydrates are shared by *A*. *fumigatus*, *C*. *auris*, *C*. *neoformans*, and *C*. *albicans*, they are present at different relative levels and are organized into distinct structural arrangements across these species (99, 124, 140).

To understand the similarities and differences between fungal CW, it is crucial to determine how their structural “bricks” are organized within the intact wall. Conventional biochemical approaches based on the solubilization and fractionation of CW components have provided fundamental insights into CW composition and polysaccharide linkages (60). However, the chemical disruption of the CW during these procedures may limit the information obtained regarding the spatial organization and molecular dynamics of its components (56). More recently, ssNMR has enabled molecular-level analysis of fungal CW architecture in intact wall preparations, reducing the need to separate wall components before structural assessment (140). By retaining the polysaccharide network *in situ*, ssNMR has shifted the interpretation of CW carbohydrates from a compositional inventory to a supramolecular model in which polymer dynamics, packing density, hydration, and proximity to neighboring wall components help define their structural and biological roles. Across WHO critical priority fungi, ssNMR has revealed a common organizational principle in which CW polysaccharides are distributed into domains with distinct physical properties, while the composition and molecular associations defining these domains vary markedly between fungi (99, 124, 141). In *C. albicans* and *C. auris*, chitin and β-1,3-glucan are enriched in less mobile wall regions, while β-1,6-glucan and a mobile β-1,3-glucan fraction contribute to more dynamic regions of the wall (124). In *A. fumigatus*, chitin and α-1,3-glucan are associated with a compact and relatively immobile wall region, while β-glucans contribute to a more hydrated polysaccharide environment (141). In *C. neoformans*, multiple structural forms of α-1,3-glucan contribute to the CW scaffold and establish distinct molecular associations with chitin, chitosan, β-glucans, melanin, and capsule-associated components (99). Taken together, these findings demonstrate that, although these fungi share several structural CW features, their cell walls are not identical, with CW architecture and component exposure varying both between species (**Table 3**) and across distinct morphotypes, thereby shaping their interactions with the host.

**Table 3:** Cell wall species-intrinsic characteristics of the WHO priority fungal pathogens.

Species	Intrinsic characteristics	Suggested function
*Aspergillus fumigatus*	Rodlets and mellanin	Hydrophobicity and immune evasion
*Cryptococcus neoformans*	GXM and chitosan	Virulence factor and stability
*Candida albicans*	Mannosylated proteins	Adhesion to epithelium and immune modulation
*Candidozyma auris*	α-1,2-manose-phosphate	Clade differentiation and resistance

Morphological transitions can be triggered by host-associated cues, including interactions with immune cells, and are accompanied by extensive remodeling of CW architecture and composition, promoting adaptation and survival within the host (33, 56, 77, 100). The resulting CW remodeling may be morphotype-dependent and can alter polysaccharide exposure at the cell surface (56, 80, 100). These morphotype-associated changes are observed across distinct fungal transitions, although the components and structural changes involved differ between species: β-glucan exposure changes during yeast–hypha morphogenesis in *C*. *albicans* (81); *C*. *auris* can undergo host-induced filamentous transitions (115); germination in *A. fumigatus* is associated with marked changes in the conidial surface (56); titanization in *C. neoformans* is accompanied by changes in both CW and capsule composition (100).

While ssNMR has added a supramolecular dimension to the classical layered model of the fungal CW by revealing rigid and mobile domains in intact cells, it remains unclear whether these architectures represent a conserved organizational principle across fungal species or are context-dependent structures shaped by species, morphotype, and environmental conditions, and to what extent they reflect the CW architecture adopted *in vivo* during host infection.

The species-specific compositional and structural signatures of the fungal CW may also provide valuable features for pathogen detection in clinical samples. Exploiting these molecular differences through analytical platforms combined with computational and *in silico* approaches may offer new opportunities to improve fungal identification and diagnosis.

## *In silico* approaches toward diagnosis breakthrough

Detectable signatures derived from CW and capsular glycans may arise from antigen release into clinical fluids, surface exposure on fungal cells, or recognition by glycan-binding molecules and antibodies. Examples of clinically relevant glycan signatures include galactomannan and β-glucan in invasive aspergillosis, mannan and β-glucan in invasive candidiasis, and cryptococcal GXM antigen in cryptococcosis (143–147). Glycan-associated signatures can be captured through complementary analytical readouts, including serological signals, mass spectrometry profiles, imaging features, glycan-binding profiles, and spectroscopic patterns. When analyzed computationally, these datasets may support more accurate detection and differentiation of fungal pathogens.

Raman and infrared spectroscopy represent spectroscopic approaches capable of linking CW biological chemistry to computational analysis. Both methods capture glycan-associated information through molecular vibrations generated by chemical bonds within fungal CW polysaccharides. In particular, C–O, C–C, C–O–C glycosidic bonds, anomeric configurations, and acetylated groups contribute to spectral features associated with glucans, chitin, and other CW carbohydrates (142, 148, 149). Therefore, differences in CW glycan composition, linkage patterns, and relative abundance can alter the overall spectral profile captured from fungal cells or clinical samples. Machine-learning models can use these multivariate spectral differences to recognize fungal molecular fingerprints, rather than relying on a single isolated marker (142). The extraction of diagnostic information from complex spectral datasets supports the use of artificial intelligence (AI) for identifying fungal structures and molecular patterns in patient samples (143, 150).

In invasive aspergillosis, for instance, blood culture tests do not always provide accurate results, which can hinder correct diagnosis and potentially impact treatment selection. In this sense, Elkadi et al. (2021) used infrared spectroscopy with machine learning approaches to identify *Aspergillus* species in blood plasma (142). The approach was able to detect constituents of the fungal CW based on infrared spectra with the aid of AI to recognize *Aspergillus* molecular fingerprints. These findings open new avenues toward searching for new methods to improve AI-assisted diagnosis, as they present high sensitivity and specificity even in the presence of drugs and other pathogens. As a culture-independent platform, it represents a faster strategy to identify CW components compared to the traditional methods, which is crucial in the initial stages of invasive aspergillosis.

More recently, Xu et al. (2023) developed a single-cell Raman spectroscopy approach aided by machine learning algorithms to successfully identify fungal components (143). By analyzing the proportion of fungal molecules, such as ergosterol, glucans, and chitin, the authors reached high accuracy in detecting fungal structures in biological samples. Impressively, using only five cells per patient, the results obtained by the authors correctly identified seven uncultured urinary tract infection samples, distinguishing *Candida* species, including *C. albicans*, *C. tropicalis, C. parapsilosis*, and *C. guilliermondii.* Despite the low number of identified samples, the findings represent a promising approach that combines high-resolution methods with artificial intelligence.

Compared with established fungal diagnostics, including culture, microscopy, antigen detection and PCR-based assays, AI-assisted platforms offer a complementary strategy rather than a replacement for current workflows. Conventional methods remain central to clinical diagnosis, but they can be limited by slow turnaround, variable sensitivity and specificity, dependence on sample type, and the need to interpret results within the clinical context (142, 143, 151–153). In contrast, AI-assisted spectroscopic and imaging-based tools can analyze multivariate molecular or morphological patterns, potentially enabling faster culture-independent detection and species-level discrimination (142, 143). However, their clinical implementation still requires larger multicenter validation studies, standardized sample-processing pipelines, model interpretability, and direct comparison with existing diagnostic assays. Future work should therefore define where AI-assisted methods add the greatest value, particularly as adjunct tools for early diagnosis, rapid species identification, and integration with established biomarkers and molecular tests.

Together, these approaches highlight the fungal CW as a source of species-specific molecular information with diagnostic value. Importantly, the same CW features that support pathogen detection also reveal structural and biochemical vulnerabilities that can be explored therapeutically. Such a connection provides a rationale for considering the fungal CW not only as a diagnostic signature, but also as a target for the development of new antifungal strategies.

## Cell wall as a target for designing new antifungal drugs

As the front line of systemic fungal infection treatment, there are four classes of antifungals available: echinocandins, polyenes, azoles, and pyrimidine analogues (154). However, the emerging multidrug resistance phenotypes of different species have become a major challenge for the treatment of fungal infections, impacting the establishment of treatment protocols (155). The development of next-generation antifungals is an advanced field aiming to design new drugs using sophisticated molecular design tools and drug delivery strategies (156).

Designing molecules with antifungal activity is not trivial and requires specific molecular targets in fungal cells (157). The fungal CW is a source of several different biomolecules associated with virulence and resistance, making them potential targets for antifungal therapy (29). The echinocandin class of antifungals inhibits β-1,3-glucan synthase, affecting the CW structure, although resistance has been reported in *Candida* species (158, 159). However, new antifungals targeting chitin, CW proteins, and β-1,3/1,6-glucans aiming to disrupt CW integrity are currently under testing (156, 157).

Molecules in current clinical trials often target CW components, acting by binding to glucan/chitin synthases or by affecting GPI anchor biosynthesis (156). Ibrexafungerp (formerly known as SCY-078) and rezafungin, for instance, are inhibitors of β-1,3-glucan synthase. Ibrexafungerp is under Phase III of clinical trials for vulvovaginal candidiasis and drug-resistant fungal infections, while rezafungin is being evaluated for invasive candidiasis (156).

Ibrexafungerp is a non-azole triterpenoid antifungal that displays an echinocandin-distinct structure, developed for treating sporadic and/or recurrent vulvovaginal candidiasis (160). As a derivative of enfumafungine, its activity relies on the inhibition of the ß-(1,3)-D-glucan synthase enzyme, being the first oral medication developed for this purpose. As ß-(1,3)-D-glucan synthase is directly involved in the synthesis of ß-(1,3)-D-glucan, this molecule represents an alternative for treating patients whose pharmacological therapies are not successfully reached with azoles (as antifungal resistance is consistently rising with azole drugs). Notably, Ibrexafungerp has presented efficacy against *C. albicans, C. glabrata, C. parapsilosis, C. krusei*, and *C. tropicalis*, showing promising efficacy in the ascension of new antifungal therapies.

In addition, nikkomycin Z acts as a dual inhibitor by mimicking uridine diphosphate N-acetylglucosamine (UDP-GlcNAc) to bind to the catalytic site and penetrate the enzyme channel, thereby preventing chitin elongation. Currently, it is under Phase II of clinical trials for coccidioidomycosis (156). Furthermore, fosmanogepix, in turn, inhibits the Gwt1 protein, interfering with the GPI anchor biosynthesis pathway. It is currently under Phase III of clinical trials for candidiasis and Phase II for other invasive fungal infections (156).

## Concluding remarks

The fungal cell wall is not merely a static protective barrier, but a dynamic and highly adaptable interface that supports fungal growth, stress adaptation, immune evasion, and host interaction. Current knowledge has revealed major differences in CW composition, organization, and remodeling across fungal species and morphotypes, highlighting the biological relevance of this structure during infection. However, important gaps remain, including how cell wall architecture changes *in vivo*, how environmental and host-associated pressures shape wall remodeling, and how these changes influence immune recognition, persistence, and antifungal tolerance. Technological advances, including ssNMR, high-resolution imaging, glycan-based detection platforms, and AI-assisted analytical approaches, have created new opportunities to investigate these questions, although broader validation, standardization, and integration with clinical workflows are still needed. Future research should therefore combine structural, biochemical, immunological, computational, and therapeutic approaches to identify conserved and species-specific vulnerabilities within the fungal cell wall. Targeting cell wall integrity and exploiting CW-derived signatures may provide new routes for the development of next-generation antifungal therapies and improved diagnostic strategies.
